# Re-Evaluating Anatomical Determinants of Foreign Body Aspiration in Adults

**DOI:** 10.3390/jcm15103913

**Published:** 2026-05-19

**Authors:** Huseyin Yildiran, Atilla Can, Zeliha Fazliogullari

**Affiliations:** 1 Department of Thoracic Surgery, Faculty of Medicine, Selcuk University, Konya 42130, Turkey; atillacan_ac@yahoo.com; 2 Department of Anatomy, Faculty of Medicine, Selcuk University, Konya 42130, Turkey; z_topal@yahoo.com

**Keywords:** foreign-body aspiration, tracheobronchial anatomy, bronchial length, airway morphology, aspiration lateralization

## Abstract

**Objectives**: Tracheobronchial foreign-body aspiration is a clinically significant condition in adults. Although classical teaching attributes the right-sided predominance to the steeper, shorter, and wider morphology of the right main bronchus, this explanation has not been empirically validated in patients with confirmed foreign-body aspiration. **Methods**: This retrospective study included adult patients (≥18 years) who underwent bronchoscopic removal of foreign bodies from the right or left main bronchus between 2012 and 2025. Only individuals with available preoperative chest radiographs were analyzed. Measurements included tracheobronchial angles, bronchial and tracheal diameters, bronchial lengths, and estimated airway volumes calculated under cylindrical assumptions. **Results**: Of 35 included patients, 26 (74.3%) had right-sided and 9 (25.7%) had left-sided bronchial foreign bodies. At the population level, the right main bronchus was significantly steeper, wider, and shorter than the left; however, these anatomical differences did not distinguish patients with right- versus left-sided foreign bodies. Tracheobronchial angles, tracheal dimensions, bronchial diameters, and diameter ratios were all similar between the two groups (*p* > 0.05). In contrast, right bronchial length and volume were significantly greater in patients with right-sided foreign bodies (*p* = 0.004 for both). Exploratory ROC analysis further suggested that a right bronchial length exceeding 23.28 mm may be associated with an increased tendency toward right-sided migration within this cohort. **Conclusions**: Increased right bronchial length and volume may facilitate a preferential pathway for airflow continuity toward the right bronchial tree. These findings challenge long-standing anatomical assumptions and highlight the need to incorporate geometric and airflow-dynamic factors into future models of aspiration mechanics.

## 1. Introduction

Tracheobronchial foreign-body aspiration remains a clinically significant problem across all age groups and can lead to life-threatening airway obstruction, recurrent infections, or delayed respiratory compromise if not promptly recognized. Although the epidemiology and clinical manifestations of aspiration differ between children and adults, foreign bodies in adult patients continue to pose diagnostic challenges due to their often subtle or nonspecific presentation [1,2]. Prior studies have described a wide variety of aspirated materials—including organic matter, bones, teeth, and metallic objects—reflecting the heterogeneity of aspiration mechanisms in the adult population [3,4]. Despite decades of clinical experience, fundamental questions regarding the determinants of foreign-body migration within the tracheobronchial tree remain unanswered.

A consistent observation across the literature is the predominance of foreign bodies in the right bronchial tree, with multiple studies reporting right-sided involvement in more than 60–70% of adult cases [4,5,6]. This pattern has traditionally been attributed to differences in airway anatomy: the right main bronchus is typically described as steeper, shorter, and slightly wider than the left, providing a more direct continuation of the tracheal axis [7,8]. Accordingly, standard anatomical teaching has long asserted that these structural characteristics naturally predispose aspirated material to enter the right main bronchus. However, despite the widespread acceptance of this explanation, there has been limited empirical investigation into whether the specific anatomical parameters of individual patients truly influence the direction of foreign-body migration.

Recent advances in imaging-based morphometry have enabled more precise quantification of tracheobronchial anatomy, yet previous studies have largely focused on descriptive measurements rather than directly evaluating anatomical differences between patients with right- versus left-sided foreign bodies. As a result, it remains unclear whether classical anatomical assumptions accurately explain the observed clinical distribution of aspirated material, or whether other variables—such as airflow dynamics, airway curvature, bronchial length, or segmental volume—may play underrecognized roles in governing aspiration pathways. Moreover, the contribution of airway geometry ratios and their potential influence on intraluminal pressure gradients have not been systematically evaluated in the context of foreign-body lateralization.

Given these knowledge gaps, a detailed reassessment of tracheobronchial morphology in patients with confirmed foreign-body aspiration is warranted. The present study seeks to address this unmet need by providing a comprehensive radiographic analysis of airway dimensions, bronchial angles, segmental lengths, and calculated airway volumes in adult patients with right- or left-sided bronchial foreign bodies. By comparing anatomical parameters between these groups, this investigation aims not only to test the validity of long-held anatomical assumptions but also to identify previously unrecognized geometric factors that may influence the direction of foreign-body migration within the adult airway.

## 2. Materials and Methods

This study included adult patients (≥18 years) who underwent bronchoscopic removal of foreign bodies from the right or left main bronchus between 2012 and 2025. Only those with available preoperative chest radiographs stored in the hospital PACS system were included. Patients younger than 18 years, those with known bronchial anomalies, cases in which the foreign body was located in the trachea, and patients whose imaging quality did not allow reliable measurements were excluded from the study.

**Figure 1:**
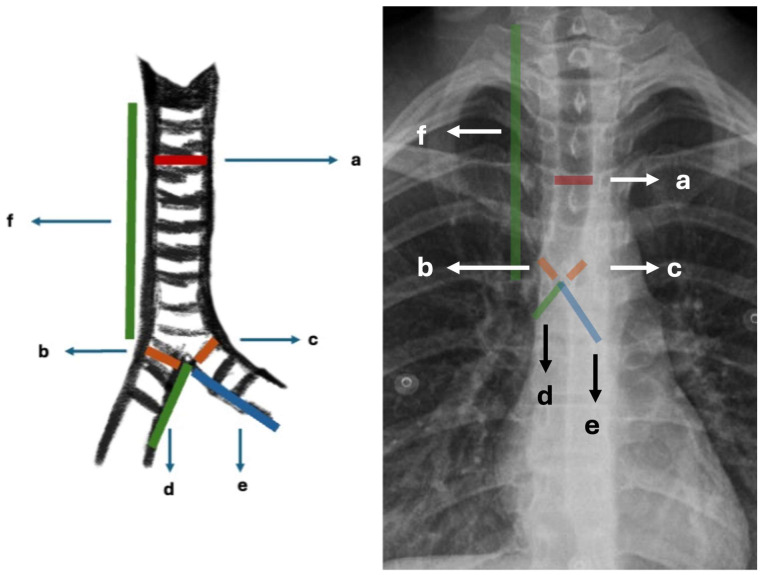
Illustrative representation of tracheobronchial measurements: (**a**) tracheal diameter: tracheal lumen diameter at the midclavicular level (mm); (**b**) right main bronchus diameter: internal lumen diameter of the right main bronchus at the level of the carina (mm); (**c**) left main bronchus diameter: internal lumen diameter of the left main bronchus at the level of the carina (mm); (**d**) right main bronchus length: distance from the level of the carina to the origin of the right upper lobe bronchus (mm); (**e**) left main bronchus length: distance from the level of the carina to the origin of the left upper lobe bronchus (mm); (**f**) tracheal length: distance from the cricoid cartilage to the level of the carina (mm) (mm: millimeters).

**Figure 2:**
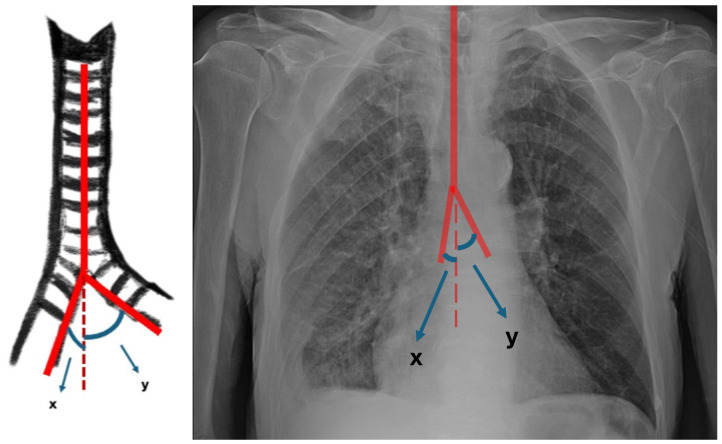
Illustrative representation of the angle measurements between the main bronchi and the trachea: (**x**) right tracheobronchial angle: the acute angle between the line following the tracheal axis and the line following the right main bronchus axis; (**y**) left tracheobronchial angle: the acute angle between the line following the tracheal axis and the line following the left main bronchus axis (angles are expressed in degrees).

Demographic data, including age and sex, as well as the type of foreign body (organic material, tooth, needle, or blood clot) and its location (right or left main bronchus), were retrieved from patient records. On preoperative chest radiographs, the diameters of the right and left main bronchi were measured at the level of the tracheal bifurcation. Tracheal diameter was measured at the linea interclavicularis level, and tracheal length was defined as the distance from the cricoid cartilage to the carina, all recorded in millimeters. The longitudinal length of each main bronchus, defined as the distance from the main carina to the origin of the upper lobe bronchus, was also measured in millimeters (Figure 1). The acute angles formed between the main bronchi (right and left) and the trachea were measured and recorded by drawing a straight line following the tracheal axis as a reference (Figure 2).

In addition, airway volumes were estimated under the assumption that each segment approximated a cylindrical structure. Using the equation V = π × r^2^ × h, volumetric calculations were performed separately for the trachea and for the right and left main bronchi.

All measurements were independently performed by two thoracic surgeons. To assess both inter- and intra-observer reliability, X-ray images from a randomly selected subset of 10 patients were re-measured one week apart by each observer. The consistency between the two observers and between repeated measurements was evaluated using the Intraclass Correlation Coefficient (ICC). Both inter-observer and intra-observer ICC values exceeded 0.90, indicating excellent measurement reliability. After confirming this high level of agreement, the mean values of the two observers’ measurements were used for the final statistical analyses. Given that measurements were derived from two-dimensional chest radiographs, potential limitations related to projectional distortion, patient positioning variability, and lack of depth resolution were considered. However, this approach reflects routine clinical practice in the evaluation of suspected tracheobronchial foreign bodies, where plain radiography is often the primary and sometimes the only available imaging modality. Volumetric estimations were performed under the simplifying assumption of cylindrical geometry. These calculations were intended for comparative analysis between groups rather than precise anatomical quantification. Given the complex, curved, and non-cylindrical nature of the tracheobronchial tree, volumetric calculations based on a cylindrical assumption should be interpreted as comparative rather than absolute measurements. All measurements were performed on anonymized imaging datasets. While the side of the foreign body may be inferable from imaging in certain cases, the high inter- and intra-observer agreement (ICC > 0.90) supports the robustness and reproducibility of the measurement process.

Statistical analyses were performed using IBM SPSS Statistics version 31.0. (IBM SPSS, Chicago, IL, USA). Comparisons between the two groups included age, sex, right and left tracheobronchial angles, diameters and lengths of the right and left main bronchi, ratios of bronchial diameters to tracheal diameter, and calculated volumes of the trachea and both main bronchi. The normality of distribution was assessed using the Shapiro–Wilk test, and homogeneity of variances was evaluated with Levene’s test. In cases where variances were unequal, Welch’s t-test was applied. For continuous variables, the independent-samples t-test was used when data were normally distributed, and the Mann–Whitney U test was used for non-normally distributed variables. The distribution of sex between the groups was analyzed using Fisher’s exact test. A *p*-value of <0.05 was considered statistically significant.

Approval for the study was obtained from the local ethics committee with the decision dated 16 December 2025, and numbered 2025/753. This study involving human participants was performed in accordance with the ethical standards of the institutional and national research committee and with the 1964 Helsinki Declaration and its later amendments or comparable ethical standards.

## 3. Results

**Figure 3:**
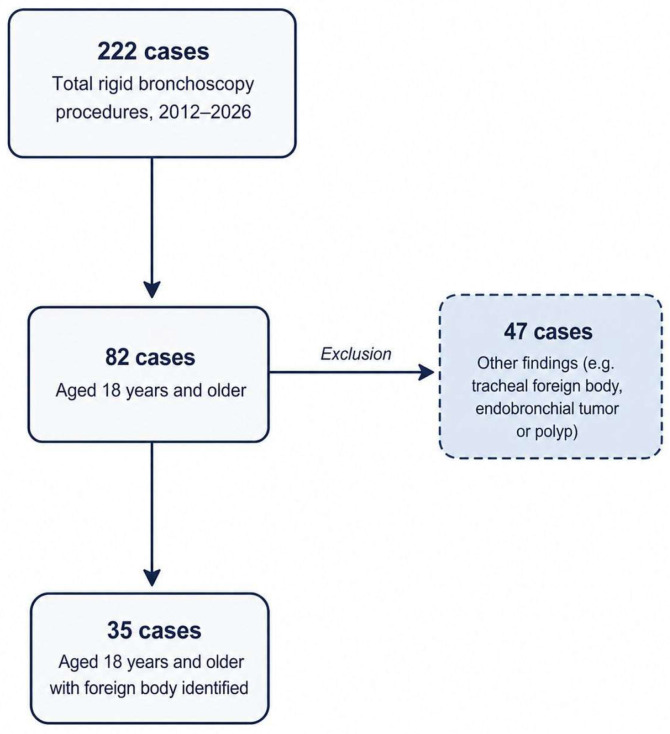
Flow diagram.

The patients included in the study were divided into two groups based on the location of the foreign body: those with foreign bodies in the right main bronchus (Group 1) and those with foreign bodies in the left main bronchus (Group 2). A total of 35 patients (16 female and 19 male) with a mean age of 60.11 ± 20.34 years were analyzed (Figure 3, Table 1). Foreign bodies were located in the right bronchial tree in 26 patients (74.28%) and in the left bronchial tree in 9 patients (25.71%). The types of foreign bodies identified were organic materials (n = 25, 71.42%), teeth (n = 5, 14.28%), needles (n = 4, 11.42%), and blood clots (n = 1, 2.85%) (Table 2).

The mean age was 63.77 ± 16.99 years in Group 1 and 49.56 ± 26.23 years in Group 2. There was no statistically significant difference in age between the two groups (t(33) = 1.872, *p* = 0.070), with a Cohen’s d of 0.724. When sex distribution was compared, Fisher’s exact test was used because more than 50% of the expected cell counts were below five. No significant difference was observed between the groups (*p* = 0.700) (Table 3).

In the overall population, the mean right and left tracheobronchial angles were 27.42 ± 7.90 and 50.71 ± 8.93 degrees, respectively, confirming that the right side is anatomically more vertical (*p* < 0.001, independent-samples t-test). However, when lateralization of the foreign body was examined, the right tracheobronchial angle did not differ between Group 1 and Group 2 (27.51 ± 9.11 vs. 27.15 ± 2.42; *p* > 0.05). Similarly, the left tracheobronchial angle showed no significant difference between the groups (50.46 ± 8.33 vs. 54.43 ± 11.02; *p* > 0.05).

When tracheal diameter was compared between the groups, the mean rank was 18.63 for Group 1 and 16.17 for Group 2. Mann–Whitney U analysis showed no statistically significant difference between the groups (*p* > 0.05). Similarly, for tracheal volume, the mean ranks were 18.58 in Group 1 and 16.33 in Group 2, with no significant difference detected (*p* > 0.05).

In the overall population, the mean diameters of the right and left main bronchi were 14.59 ± 3.07 mm and 10.98 ± 2.56 mm, respectively, confirming that the right bronchus is significantly wider (*p* < 0.001). However, when lateralization of the foreign body was analyzed, the right bronchial diameter did not differ between Group 1 and Group 2 (14.86 ± 3.09 vs. 13.83 ± 3.05; *p* > 0.05). The left bronchial diameter likewise showed no significant difference between the groups (11.22 ± 2.67 vs. 10.29 ± 2.21; *p* > 0.05).

In the overall population, the ratios of the right and left bronchial radii to the tracheal radius were 0.86 ± 0.09 and 0.65 ± 0.11, respectively, demonstrating a significant difference between the two sides (*p* < 0.05). However, when foreign-body lateralization was considered, no significant differences were observed. The ratio of the right bronchial radius to the tracheal radius was 0.87 ± 0.09 in Group 1 and 0.82 ± 0.07 in Group 2, while the ratio for the left bronchus was 0.66 ± 0.11 in Group 1 and 0.62 ± 0.10 in Group 2 (all *p* > 0.05).

In the overall sample, the mean lengths of the right and left main bronchi were 26.09 ± 5.99 mm and 46.35 ± 8.89 mm, confirming that the right bronchus is significantly shorter (*p* < 0.001). When analyzed by lateralization, the right bronchial length was 27.73 ± 5.40 mm in Group 1 and 21.36 ± 5.24 mm in Group 2. The right bronchus was significantly longer in patients with right-sided foreign bodies compared with those with left-sided foreign bodies (27.73 ± 5.40 mm vs. 21.36 ± 5.24 mm; *p* = 0.004), a difference supported by a large effect size (Cohen’s d = 1.186). In contrast, left bronchial length did not differ significantly between the groups (47.68 ± 8.31 mm vs. 42.52 ± 9.87 mm; *p* > 0.05) (Table 4).

In the overall population, the right bronchial volume was significantly greater than the left bronchial volume, consistent with the larger caliber and longer longitudinal extension of the right main bronchus (*p* < 0.05).

However, when patients were stratified according to foreign-body lateralization, individuals with right-sided foreign bodies demonstrated significantly greater right bronchial volumes compared with those with left-sided foreign bodies (21,062.91 ± 11,258.38 mm^3^ vs. 12,920.94 ± 5275.53 mm^3^; *p* = 0.004), supported by a medium-to-large effect size (Cohen’s d = 0.803). No significant difference was observed for left bronchial volume between the groups (20,319.97 ± 10,335.32 mm^3^ vs. 15,191.84 ± 8970.29 mm^3^; *p* > 0.05).

According to ROC curve analysis, right bronchial length demonstrated the strongest discriminatory ability for predicting the direction of foreign-body migration (AUC = 0.803, 95% CI: 0.647–0.959, *p* < 0.001). In this exploratory cohort, a right bronchial length value above 23.28 mm demonstrated a sensitivity of 80.8% and a specificity of 77.8% for distinguishing right-sided aspiration patterns. (Youden Index = 0.432). Consistent with this finding, the AUC for right bronchial volume was 0.709 (*p* = 0.004), indicating moderate accuracy in distinguishing the aspiration direction. In contrast, left bronchial length exhibited poor discriminatory power for predicting lateralization of the foreign body.

## 4. Discussion

Tracheobronchial foreign-body aspiration remains a clinically important condition across all age groups; however, the mechanism, localization, and management of aspiration in adults differ substantially from those in children. Although the highest incidence is reported in children under three years of age, foreign-body aspiration can occur at any age. Mise et al. reported a mean age of 64.8 years in adult series, which is consistent with the mean age of 60.11 years observed in our study [9]. The tendency for aspiration to occur more frequently in older adults may be attributed to age-related weakening of protective reflexes such as coughing, decreased oral sensation due to tooth loss or denture use, and the higher prevalence of comorbid conditions associated with dysphagia. Additionally, medications commonly used in older populations—including sedatives, antihypertensives, and anticholinergic agents—may suppress reflexes, cause xerostomia, and further impair swallowing mechanisms, collectively increasing the risk of aspiration. These factors likely account for the predominance of older age groups in both our cohort and previously published adult series.

Bones, organic materials, and inorganic objects are reported as the most common tracheobronchial foreign bodies in adult patients [10]. In our series, organic materials constituted the majority of aspirated objects, consistent with previously published data. Age and sex were comparable between the two groups, providing baseline standardization and allowing us to assume that subsequent statistical comparisons between groups were methodologically reliable (Group 1 vs. Group 2).

Bronchial foreign bodies have been reported in the literature to involve the left bronchial tree in approximately 25–40% of cases [1,11,12]. The 25.71% rate of left-sided aspiration observed in our study aligns with these published figures, further supporting the well-established clinical observation that foreign-body aspiration in adults predominantly affects the right side.

This study also confirmed that the right tracheobronchial angle is anatomically steeper than the left. However, no statistically significant relationship was identified between individual angle variations and the direction of foreign-body migration. The right tracheobronchial angle was similar in patients whose foreign bodies entered the right bronchial tree and in those whose foreign bodies entered the left. Likewise, the left tracheobronchial angle did not differ between these groups. In other words, aspirated objects did not preferentially enter the right bronchus simply because it diverges at a steeper angle; the angle measurements remained remarkably stable across patients regardless of aspiration direction.

In contrast, the prevailing literature asserts that, among adults, aspirated foreign bodies preferentially lodge in the right bronchial tree—particularly in the bronchus intermedius and right lower lobe—and attributes this pattern to the right main bronchus being more vertical, shorter, and slightly wider than the left. Multiple studies consistently report a right-sided predominance, with 61–81% of foreign bodies identified in the right bronchial tree [13,14]. The commonly proposed explanation is that the right main bronchus is inherently steeper and marginally wider, providing a more direct continuation of the tracheal axis for aspirated objects [7,15,16].

Classical anatomical explanations, particularly the steeper and wider configuration of the right main bronchus, remain well supported at the population level. However, our findings suggest that these parameters alone may not fully explain individual variability in aspiration patterns in adults.

However, in our study, the right tracheobronchial angle was nearly identical in patients with right- and left-sided foreign bodies, indicating that angle alone does not determine lateralization. These findings suggest that the well-established tendency for foreign bodies to lodge on the right side cannot be explained solely by tracheobronchial angle anatomy. Instead, the mechanism is likely multifactorial, involving a more complex and dynamic interplay of anatomical and aerodynamic influences. Importantly, these findings do not contradict the well-established population-level anatomical tendency toward right-sided aspiration; rather, they indicate that such anatomical features alone are insufficient to explain individual variability in foreign-body lateralization.

In our study, the right main bronchus was indeed wider than the left, consistent with classic anatomical descriptions. However, when patients with right- versus left-sided foreign bodies were directly compared, bronchial diameters on both sides were statistically indistinguishable between the two groups. Thus, contrary to long-held assumptions and widely propagated claims in the literature, the predominance of right-sided aspirations cannot be attributed to bronchial caliber. Although the right bronchial radius is typically larger than that of the left—much like its steeper divergence from the trachea—this structural feature did not influence whether a foreign body ultimately entered the right or left bronchial tree.

Similarly, tracheal diameter did not differ between patients with right- and left-sided foreign bodies. This raised the question of whether the proportional relationships between tracheal and bronchial diameters might play a role by altering local pressure gradients. Indeed, population-level analyses confirmed that the ratios of bronchial-to-tracheal radii differ significantly between the right and left sides. Nevertheless, when stratified by the direction of foreign-body migration, these ratios were nearly identical in both groups. In other words, neither absolute bronchial diameter nor its proportional relationship to the trachea predicted the lateralization of foreign-body aspiration.

Collectively, these findings indicate that the classical explanation—attributing right-sided predominance to a wider and more vertical right bronchus—is insufficient. Bronchial diameter, whether considered in isolation or relative to the trachea, does not determine aspiration direction. The mechanism underlying the right-sided bias is therefore more complex than previously assumed and likely involves dynamic airflow characteristics rather than static luminal geometry.

In our study, the right main bronchus was found to be substantially shorter than the left when evaluated across the entire cohort, consistent with established anatomical descriptions. However, when patients were stratified by the direction of foreign-body migration, a striking pattern emerged: individuals with right-sided foreign bodies had significantly *longer* right main bronchi compared with those whose foreign bodies had migrated to the left. A parallel finding was observed in volumetric assessments, whereby right-sided aspiration was associated with a markedly greater right bronchial volume.

It is important to note that the present study demonstrates associations rather than causality. The cross-sectional design does not allow direct assessment of the mechanisms underlying foreign-body migration. These observations may reflect underlying airflow-related phenomena; however, such interpretations remain speculative and require validation through dedicated functional and computational studies.

These findings may reflect a possible physiological association. A longer right main bronchus may be associated with altered airflow distribution patterns within the tracheobronchial tree. Although the present study did not directly evaluate airflow dynamics, this geometric configuration may reflect conditions that favor preferential migration toward the right bronchial system. These interpretations should nevertheless be considered speculative and hypothesis-generating. It should be noted that airway geometry was assessed using two-dimensional radiographic measurements. Given the inherently curved and non-uniform structure of the tracheobronchial tree, these measurements represent simplified approximations. Therefore, absolute geometric values should be interpreted cautiously.

In other words, increased right bronchial length and volume may be associated with “a preferential migration tendency” for airflow continuity toward the right bronchial tree. Importantly, the present study demonstrates associations between bronchial geometry and foreign-body lateralization; however, it does not establish causal mechanisms. The proposed airflow-related explanations should therefore be interpreted as hypothesis-generating. Foreign-body aspiration is likely a multifactorial process influenced not only by airway geometry but also by dynamic and situational factors. Body position at the time of aspiration; the size, shape, and weight of the foreign body; and cough dynamics and airflow patterns may all contribute to the direction of migration. In addition, anatomical variations such as carinal orientation and subtle differences in airway branching may further influence aspiration pathways. These factors were not directly assessed in the present study and may partially explain the variability observed in foreign-body localization.

In our exploratory analysis, a right main bronchial length exceeding 23.28 mm was associated with a higher likelihood of right-sided aspiration within this cohort. However, this threshold was internally derived from a relatively small retrospective sample and should not be interpreted as a clinically validated cutoff. External validation in larger, ideally CT-based, prospective cohorts is required before any clinical applicability can be considered.

A major limitation of our study is that the inclusion of only patients with available preoperative chest radiographs may introduce selection bias, potentially favoring specific clinical presentations or types of foreign bodies, and may limit the generalizability of the findings. Another important limitation is the retrospective design of the study, which precludes access to detailed clinical information regarding the circumstances of aspiration, such as body position at the time of the event, cough dynamics, or the exact mechanism of inhalation. In addition, the absence of three-dimensional imaging and airflow analysis represents a critical limitation. Airway geometry was assessed using two-dimensional radiographic measurements, and no computational fluid dynamics or functional airflow modeling was performed. Therefore, the proposed mechanistic interpretations regarding airflow behavior and preferential pathways remain speculative. Future studies incorporating high-resolution 3D imaging and computational modeling are required to validate these hypotheses.

From a clinical perspective, these findings suggest that while population-level anatomy supports a right-sided predominance of aspiration, individual anatomical variations may influence the actual direction of foreign-body migration. In selected cases where detailed imaging is available, parameters such as bronchial length may provide additional insight into aspiration patterns and procedural planning. Although these observations are not yet sufficient to guide routine clinical decision-making, they may contribute to a more individualized understanding of airway anatomy in the context of foreign-body aspiration.

Future research should aim to validate these findings using larger, prospective cohorts with standardized imaging protocols. In particular, high-resolution three-dimensional CT-based analyses would facilitate more accurate characterization of airway geometry, including curvature, tapering, and spatial relationships that cannot be captured by two-dimensional radiographs. Furthermore, integration of computational fluid dynamics modeling may provide deeper insight into airflow behavior and its interaction with airway structure, potentially clarifying the mechanisms underlying preferential migration pathways. Finally, extending analyses to segmental and subsegmental bronchial levels may further refine our understanding of foreign-body distribution within the tracheobronchial tree.

## 5. Conclusions

In conclusion, our findings suggest that classical anatomical explanations for the right-sided predominance of tracheobronchial foreign-body aspiration in adults may not fully account for individual patterns of foreign-body migration. Although the right main bronchus is anatomically steeper and wider at the population level, these parameters did not differ between patients with right- and left-sided foreign bodies, indicating that static airway geometry alone may not determine lateralization. Instead, our findings reveal that increased right bronchial length and volume—particularly a right bronchial length exceeding 23.28 mm—are associated with a higher probability of right-sided aspiration, suggesting the presence of a previously unrecognized preferential flow pathway. However, these findings should be interpreted as exploratory and hypothesis-generating. Further studies with larger patient cohorts, three-dimensional imaging, and computational airflow modeling are required to validate these observations and to better define the mechanisms underlying tracheobronchial foreign-body migration.

## Figures and Tables

**Table 1:** Characteristics of patients.

			Total
	Gender		
	Female	Male	
n, %	16 (45.71%)	19 (54.29%)	35 (100%)
	**Age**		
Year ± SD	49.18 ± 20.51	69.31 ± 15.35	60.11 ± 20.34

**Table 2:** The types of tracheobronchial foreign bodies.

	Foreign Body (n, %)
Organic material	25 (71.42%)
Tooth	5 (14.28%)
Needle	4 (11.42%)
Blood clot	1 (2.85%)
**Total**	35 (100%)

**Table 3:** Summary of patient data by study group.

	Right Main Bronchus (*Group 1*)	Left Main Bronchus (*Group 2*)	Total
n, %	26 (74.28%)	9 (25.72%)	35 (100%)
	**Gender**		
Female (n, %)	11 (31.43%)	5 (14.29%)	16 (45.71%)
Male (n, %)	15 (42.85%)	4 (11.43%)	19 (54.29%)
	**Age**		
Year ± SD	63.77 ± 16.99	49.56 ± 26.23	60.11 ± 20.34

**Table 4:** Morphometric measurements of the trachea and main bronchi in Group 1 and Group 2.

		Group 1	Group 2	Total	*p*-Value	Cohen’s d
Diameter (mm)	Trachea	17.01 ± 3.63	16.81 ± 4.25	16.96 ± 3.73	>0.05	-
	Right main bronchus	14.86 ± 3.09	13.83 ± 3.05	14.59 ± 3.07	<0.001	-
	Left main bronchus	11.22 ± 2.67	10.29 ± 2.21	10.98 ± 2.56		
Length (mm)	Trachea	125.27 ± 14.48	124.2 ± 15.71	125 ± 14.58	>0.05	-
	Right main bronchus	27.73 ± 5.40	21.36 ± 5.24	26.09 ± 5.99	0.004	1.186
	Left main bronchus	47.68 ± 8.31	42.52 ± 9.87	46.35 ± 8.89	>0.05	-
Angle (degree)	Right	27.51 ± 9.11	27.15 ± 2.42	27.42 ± 7.90	<0.001	-
	Left	50.46 ± 8.33	54.43 ± 11.02	50.71 ± 8.93		
Volume (mm^3^)	Trachea	122,020.13 ± 59,425.77	121,114.69 ± 78,439.03	121,787.30 ± 63,596.26	>0.05	-
	Right main bronchus	21,062.91 ± 11,258.38	12,920.94 ± 5275.53	18,969.26 ± 10,619.95	0.004	0.803
	Left main bronchus	20,319.97 ± 10,335.32	15,191.84 ± 8970.29	19,001.31 ± 10,131.52	>0.05	-
*p*-value		>0.05				
Ratio	Right main bronchus/Trachea	0.87 ± 0.09	0.82 ± 0.07	0.86 ± 0.09	>0.05	-
	Left main bronchus/Trachea	0.66 ± 0.11	0.62 ± 0.10	0.65 ± 0.11	>0.05	-
*p*-value		<0.05				

## Data Availability

The raw data supporting the conclusions of this article will be made available by the authors on request.

## References

[1] Hewlett J.C., Rickman O.B., Lentz R.J., Prakash U.B., Maldonado F. (2019). Foreign body aspiration in adult airways: Therapeutic approach. J. Thorac. Dis..

[2] Shostak E. (2018). Foreign body removal in children and adults: Review of available techniques and emerging technologies. AME Med. J..

[3] Sehgal I.S., Dhooria S., Ram B., Singh N., Aggarwal A.N., Gupta D., Behera D., Agarwal R. (2015). Foreign Body Inhalation in the Adult Population: Experience of 25,998 Bronchoscopies and Systematic Review of the Literature. Respir. Care.

[4] Jang G., Song J.W., Kim H.J., Kim E.J., Jang J.G., Cha S.-I. (2022). Foreign-body aspiration into the lower airways in adults; multicenter study. PLoS ONE.

[5] Baharloo F., Veyckemans F., Francis C., Biettlot M.P., Rodenstein D.O. (1999). Tracheobronchial foreign bodies: Presentation and management in children and adults. Chest.

[6] Sancho-Chust J.N., Molina V., Vañes S., Pulido A.M., Maestre L., Chiner E. (2020). Utility of Flexible Bronchoscopy for Airway Foreign Bodies Removal in Adults. J. Clin. Med..

[7] Tahir N., Ramsden W.H., Stringer M.D. (2009). Tracheobronchial anatomy and the distribution of inhaled foreign bodies in children. Eur. J. Pediatr..

[8] Mi W., Zhang C., Wang H., Cao J., Li C., Yang L., Guo F., Wang X., Yang T. (2015). Measurement and analysis of the tracheobronchial tree in Chinese population using computed tomography. PLoS ONE.

[9] Mise K., Jurcev Savicevic A., Pavlov N., Jankovic S. (2009). Removal of tracheobronchial foreign bodies in adults using flexible bronchoscopy: Experience 1995–2006. Surg. Endosc..

[10] Dong Y.C., Zhou G.W., Bai C., Huang H.D., Sun Q.Y., Huang Y., Han Y.P., Li Q. (2012). Removal of tracheobronchial foreign bodies in adults using a flexible bronchoscope: Experience with 200 cases in China. Intern. Med..

[11] Pinto A., Scaglione M., Pinto F., Guidi G., Pepe M., Del Prato B., Grassi R., Romano L. (2006). Tracheobronchial aspiration of foreign bodies: Current indications for emergency plain chest radiography. Radiol. Med..

[12] Ramos M.B., Fernández-Villar A., Rivo J.E., Leiro V., García-Fontán E., Botana M.I., Torres M.L., Cañizares M.A. (2009). Extraction of airway foreign bodies in adults: Experience from 1987–2008. Interact. Cardiovasc. Thorac. Surg..

[13] Moura e Sá J., Oliveira A., Caiado A., Neves S., Barroso A., Almeida J., Ferraz J.M. (2006). Tracheobronchial foreign bodies in adults—Experience of the Bronchology Unit of Centro Hospitalar de Vila Nova de Gaia. Rev. Port. Pneumol..

[14] Donado Uña J.R., de Miguel Poch E., Casado López M.E., Alfaro Abreu J.J. (1998). La fibrobroncoscopia en la extracción de cuerpos extraños traqueobronquiales en adultos [Fiber optic bronchoscopy in extraction of tracheo-bronchial foreign bodies in adults]. Arch. Bronconeumol..

[15] Kathuria B., Arora V., Wadhera R., Singh S. (2017). Sharpener blade: An unusual tracheobronchial sharp foreign body in a child. Lung India.

[16] Lowe D., Russell R.I. (1984). Tracheobronchial foreign bodies—The position of the carina. J. Laryngol. Otol..

